# A comparative analysis of vibrational spectra for odorant classification

**DOI:** 10.1371/journal.pone.0342845

**Published:** 2026-02-26

**Authors:** Andrés Álvarez-García, Georgina Rodríguez-Contreras, Penélope Rodríguez-Zamora, Ignacio L. Garzón

**Affiliations:** 1 Departamento de Física, Facultad de Ciencias, Universidad Nacional Autónoma de México, CDMX, Mexico; 2 Instituto de Física, Universidad Nacional Autónoma de México, Ciudad de México, Mexico; University of Calgary, CANADA

## Abstract

The olfactory characteristics of flowers have diverse biological and industrial applications. The physicochemical properties of odorants can reveal information about the molecules responsible for a flower’s scent. In particular, the distinct molecular characteristics are revealed by vibrational spectra in the fingerprint region. In this study, we calculate the vibrational spectra of compounds from two flower families: Orchidaceae and Apocynaceae. We classified these molecules based on their vibrational spectra using a spectral clustering algorithm and analyzed the most representative vibrational modes. This method effectively clusters molecules with distinctive odors, such as garlic, decay, and sweetness, which are associated with compounds containing heteroatoms (N and S) or conjugated systems. The clustering obtained by infrared spectroscopy reflected a better classification by compound odor compared to Raman spectroscopy and vibrational density of states. Hence, some vibrational modes, particularly those associated with specific functional groups, may play a major role in odor discrimination. These findings suggest that vibrational spectra can offer odor-relevant information for olfactory categorization.

## Introduction

Plants produce low molecular weight compounds that emit odor, known as odorants, with the highest concentration found in flowers [1]. The aroma of flowers serves various purposes, including attracting pollinators, supporting reproduction, and providing protection [2,3]. These floral emissions contain a variety of molecules, encompassing terpenoids, fatty acid derivatives, and amino acids [4,5]. For instance, some orchids emit a fruity odor caused by terpenes, aldehydes, and esters [6]. Odorant compounds are present in numerous products, such as cosmetics, flavourings, and perfumes [7].

Designing scent molecules is a labor-intensive and time-consuming process. As a result, recent olfactory research has shifted focus toward predicting the odor of new molecules, designing fragrances with specific scents, and analyzing human olfactory preferences [8]. Physicochemical parameters are used to develop and train models that identify and predict a substance’s olfactory properties and classification [9–12]. Among these parameters, vibrational spectroscopy stands out for its fingerprint region, which enables the extraction of relevant molecular information. Furthermore, molecular vibrations have attracted the attention of the scientific community, as evidence increasingly supports a vibrational theory of smell [13].

The theory of vibration suggests that olfactory receptors (ORs) can detect the unique vibrations of odorant molecules, which the brain then interprets as specific odors [14]. Luca Turin’s theory posits that olfaction operates as a spectral sense. He proposes that an inelastic electron tunneling mechanism allows olfactory receptors to detect vibrations. The signaling event depends on successful electron tunneling from a donor (D) state to an acceptor (A) state. This process is facilitated by the odorant, as the energy difference between these states aligns with a vibrational mode in the molecule [13].

There is some evidence supporting Turin’s theory based on studies using vibrational spectroscopy. Schulten and coworkers found that high tunneling rate values correspond to the vibrational modes with higher IR activity [15,16]. This result aligns with the observation that some odorless molecules, such as H_2_, N_2_, and O_2_, also show no infrared activity. These molecules belong to the D*_∞h_* point group. Since they are homonuclear, they do not display a permanent dipole moment. In addition, evidence supporting the vibrational theory includes the effect of deuteration on the sense of smell. Haffenden et al. demonstrated through sensory analysis and IR spectra that certain shifts in vibrational modes correspond to changes in scent [17]. In addition, studies have demonstrated that *Drosophila melanogaster* (fruit fly) can distinguish between deuterated and regular odorant compounds despite the absence of structural differences [18]. Finally, it has been shown that deuteration influences both the olfactory discrimination landscape and the electron tunneling rate [19].

Therefore, understanding the vibrational spectra of odorant compounds and their relationship to odors is crucial. Pandey et al. developed a vibrational classification based on the eigenvalue vibrational pseudo-spectra of the odorants. This pseudo-spectrum represents the vibrational density of states (VDOS) of each odorant. They employed a clustering algorithm to classify the vibrational modes of the VDOS, which facilitated the identification of characteristic regions associated with different molecules [20]. Ameta et al. found a strong correlation between the olfactory characteristics of molecules and their infrared spectra. They developed a multi-label classification system using advanced deep learning techniques [10,21]. However, no study has yet compared the effectiveness of different vibrational spectroscopies in classifying odorant compounds.

In this study, we calculate the IR, Raman, and VDOS spectra for a set of odorants belonging to two distinct flower families: Orchidaceae, known for sweet odors, and Apocynaceae, associated with unpleasant odors. We classify the odorants based on their vibrational spectra and analyze their characteristic frequencies. Although subtle differences in aroma between odorant compounds are not easily distinguishable, our results suggest that, by accentuating local aspects, such as functional group vibrations, IR spectroscopy demonstrates a good association between molecular vibrational spectra and perceptual odours. In contrast, the Raman and VDOS show only a slight relationship with the aroma.

## Materials and methods

We selected two flower families, Orchidaceae and Apocynaceae, to examine the relationship between the vibrational properties and the odor of their compounds. From each family, we chose three flowers, and our study was based on experimental reports that provided detailed quantification and characterization of their major compounds [22–25]. All calculations were performed using Density Functional Theory (DFT), as implemented in the Gaussian 16 electronic structure code [26]. The odorant structures were optimized using the B3LYP functional [27] and the Def2-TZVP basis set [28,29]. The frequencies were calculated from the Hessian matrix using the harmonic approximation. We calculated some vibrational spectroscopies, such as infrared and Raman, and analyzed the distribution of vibrational modes using the density of vibrational states (VDOS). The VDOS was determined by employing a Gaussian broadening of 10 cm^−1^ to the 3N–6 eigenvalues obtained from the diagonalization of the dynamical matrix [30]. This Gaussian broadening parameter has been used in previous studies of vibrational spectra because it provides good resolution for analyzing molecular vibrations in the fingerprint region [10]. Furthermore, the eigenvectors of the mass-weighted Hessian matrix correspond to the individual normal modes. Each mode was examined to identify dominant atomic motions, such as stretching, bending, and wagging.

**Fig 1:**
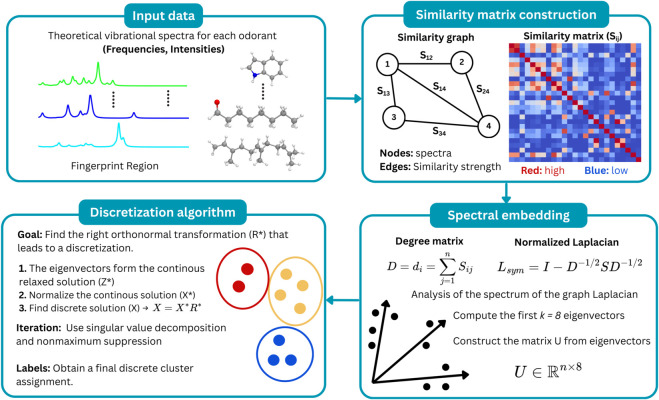
Schematic representation of the spectral clustering applied to the vibrational spectra of odorant compounds.

The spectral clustering algorithm was used to group vibrational spectra with similar profiles (Fig 1). This methodology does not make assumptions about the shapes of the clusters. In contrast to traditional clustering algorithms, such as k-means, which invariably result in clusters with convex geometric shapes [31]. The input data consists of theoretical vibrational spectra for each odorant obtained from DFT calculations. The first step involves constructing a similarity matrix graph for the data, where nodes represent the vibrational spectra and edges indicate the degree of similarity (S_*ij*_), as measured by Pearson’s correlation coefficient [32]. The similarity matrix is constructed by calculating the Pearson correlation coefficient between pairs of vibrational spectra. Each spectrum is treated as a vector of intensities sampled over the fingerprint region. Pearson’s correlation coefficient between two vibrational spectra was calculated by subtracting the mean intensity of each spectrum, multiplying the resulting deviations pointwise across the frequency grid, summing over all frequency points, and normalizing by the product of the corresponding standard deviations. This information can be visualized as a heat map of the similarity matrix (S4–S6 Figs). Then, we calculated the symmetric normalized Laplacian (*L*_*sym*_):

$$L_{sym}=I-D^{-1/2}SD^{-1/2}$$ (1)

Where D is the degree matrix and S is the similarity matrix. The degree matrix is defined as the diagonal matrix where each diagonal element is the sum of similarities for that node ($d_{i}=\sum_{j=1}^{n}S_{ij}$). The spectrum of the graph Laplacian is then analyzed, thus giving rise to the term “spectral clustering” [33].

The vibrational spectra of odorants were grouped, extracting the first eigenvectors of the normalized Laplacian. Consequently, the data are embedded in a low-dimensional space ($U\in {\mathbb{R}}^{n\times k}$), thereby facilitating the separation of clusters. In addition, we employed a discretization algorithm to identify the clusters from the eigenvectors of the Laplacian graph [34]. This implementation normalizes a continuous solution and then iteratively discretizes using singular value decomposition and non-maximum suppression. This procedure enables the optimal orthonormal transformation for the discrete solution and the corresponding cluster assignment. To determine the most effective number of clusters for the vibrational spectra, we calculated the Silhouette coefficient [35]. This metric evaluates how well each data point fits into its assigned cluster compared to other clusters, based on the distances between points [36]. Finally, the adjusted mutual information (AMI) was calculated to evaluate the relationship between the vibrational spectra and the odor descriptor for the compounds [37]. This metric is adjusted for chance, meaning that the observed similarity is due to the dataset’s structure rather than random occurrence [38,39]. Notably, the differences remain significant even with a sample size of 25 compounds. If the data’s configuration aligns with a random distribution, the AMI score will be near zero. Conversely, scores approaching 1.0 signify strong cluster agreement [40].

## Results and discussion

### Description of the aroma and chemical composition of the flowers

The Orchidaceae is a group of plants that exhibit a wide range of colors, scents, and morphologies. The species of *Maxillaria tenuifolia*, *Dendrophylax lindenii*, and *Catasetum arietinum* were selected for their sweet aroma with fruity tones. The fragrance of these plants consists mainly of terpenes, with aromatic compounds contributing secondarily. Sadler et al. examined the aroma composition of *D. lindenii*, known for its sweet floral smelling [22]. The principal odorant is farnesene (70.7 %), which possesses an apple odor [41] (Table 1). Furthermore, the impact of this compound is notable due to its concentration and low odor threshold of 0.087 ppm [42]. Ocimene, methyl salicylate, and linalool contribute minor woody and floral tones to the plant’s aroma.

**Table 1:** Odor description and relative content (%) of aroma-active compounds in the Orchidaceae and Apocynaceae families. The plant names are abbreviated as follows: *D. lin.* (*Dendrophylax lindenii*), *M. ten.* (*Maxillaria tenuifolia*), *C. ari.* (*Catasetum arietinum*), *H. ken.* (*Huernia keniensis*), *P. cub.* (*Pseudolithos cubiformis*), and *O. var.* (*Orbea variegata*).

No.	Compounds	Odor descriptor [45]	Orchidaceae family [22–24]			Apocynaceae family [25]		
			D. lin.	M. ten.	C. ari.	H. ken.	P. cub.	O. var.
1	(E,E)-*α*-farnesene	Fruit	70.7	–	24.3	–	–	–
2	(E)-*β*-ocimene	Wood	9.1	–	–	–	–	–
3	Methyl salicylate	Sweet	7.8	–	–	–	–	–
4	Linalool	Flower	4.7	–	–	–	–	–
5	(-)-*β*-Caryophyllene	Spices	–	69.0	–	–	–	–
6	(-)-*α*-Copaene	Wood	–	10.3	–	–	–	–
7	Caryophylladienol II	Spices	–	7.4	–	–	–	–
8	(Z)-Methyl-p-methoxycinnamate	Spices	–	–	18.6	–	–	–
9	*β*-Bisabolene	Wood	–	–	10.9	–	–	–
10	(E)-*α*-Bergamotene	Fruit	–	–	10.4	–	–	–
11	6-Methylhept-5-en-2-one	Fruit	–	–	4.5	–	–	–
12	(E)-Methyl-p-methoxycinnamate	Spices	–	–	3.9	–	–	–
13	(E)-Geranyl geraniol	Flower	–	–	3.3	–	–	–
14	Eucalyptol	Spices	–	–	3.2	–	–	–
15	Dimethyl disulfide	Garlic	–	–	–	22.7	10.8	–
16	Benzoic acid	Sweet	–	–	–	22.3	–	17.4
17	Octanal	Decayed	–	–	–	11.4	15.7	9.2
18	Acetophenone	Sweet	–	–	–	6.9	–	11.2
19	3-Methyl-2-pentanone	Sweet	–	–	–	6.2	–	4.2
20	Dimethyl trisulfide	Garlic	–	–	–	6.0	53.7	26.3
21	Heptanal	Decayed	–	–	–	4.8	–	–
22	Decanoic acid	Decayed	–	–	–	3.5	–	8.0
23	Benzaldehyde	Sweet	–	–	–	–	9.3	–
24	d-Limonene	Fruit	–	–	–	–	3.5	–
25	Indole	Decayed	–	–	–	–	–	5.7

The plant *M. tenuifolia* is colloquially known as the “coconut-orchid” due to its distinctive coconut-like scent. Kim et al. identified that *β*-caryophyllene is the most prevalent compound in floral organs at the full flowering stage (69.0 %) [23]. *β*-caryophyllene has a clove-like aroma and a low odor threshold (0.064 ppm) [43]. The remaining compounds contribute in a minor portion, with copaene and a derivative of caryophyllene accounting for more than 5 %. Finally, the *C. arietinum* plant has a licorice scent. It contains a greater variability of compounds, although farnesene has a slightly predominant character (24.3 %). Other significant compounds include (Z)-methyl-p-methoxycinnamate (18.6 %), *β*-bisabolene (10.9 %), and (E)-*α*-bergamotene (10.4 %) [24].

The second family is Apocynaceae, where species *Huernia keniensis*, *Pseudolithos cubiformis*, and *Orbea variegata* were selected. These species have a foul odor, which serves the function of attracting potential pollinating insects [44]. The smell of these plants is primarily composed of sulfur compounds, followed by carboxylic acids and aldehydes (Table 1). Dimethyl disulfide and dimethyl trisulfide possess an alliaceous aroma (resembling garlic or onion) [25,41]. It is also noteworthy that these sulfur compounds have a very low odor threshold, making their contribution to the overall odor profile significant [42]. Other compounds with a foul odor, including decanoic acid, indole, octanal, and heptanal, could also contribute to the plants’ aroma. The aromatic compounds, such as benzoic acid, benzaldehyde, and acetophenone, possess sweet odors, although their contribution is relatively minor [43].

Finally, we categorized the scent of the compounds using descriptors extracted from the literature to evaluate the effectiveness of the clustering based on the vibrational spectra [45]. The 25 molecules were labeled according to their associated odors: fruit, wood, sweet, spice, flower, garlic, and decayed (Table 1). This approach seeks to establish a link between the clusters formed through the vibrational spectra and the corresponding odors of the molecules.

### Infrared spectra

Vibrational spectra of the optimized structures of the odorant compounds were calculated. The infrared spectrum can be divided into three regions: the low-frequency region (below 500 cm^−1^), the fingerprint region (600–1800 cm^−1^), and the high-frequency region (2000–4000 cm^−1^). Previous studies have investigated the vibrations of compounds by C–H and C–D stretching in the high-frequency region [46]. While this analysis is valuable for monitoring the deuteration process, it does not provide any specific information about the molecule itself [47,48]. The fingerprint region is meaningful because it contains the structural characteristics, functional groups, and various bending and stretching patterns that create a unique signature for the molecule [49]. Therefore, our vibrational analysis focused on the fingerprint region of the vibrational spectra.

We compared the theoretical and experimental spectra of three compounds: linalool, benzaldehyde, and *β*-caryophyllene (S1 Fig). The calculated vibrations exhibit an overestimation of approximately 50 cm^−1^, which is a characteristic shift for DFT calculations [50]. This shift was applied to the theoretical spectra. In addition, the IR activity depends on changes in the electric dipole moment of the system with respect to the normal mode coordinates. Our calculated spectra align well with the experimental profile, demonstrating a strong agreement in the relative intensities observed in the IR spectrum [51,52].

**Fig 2:**
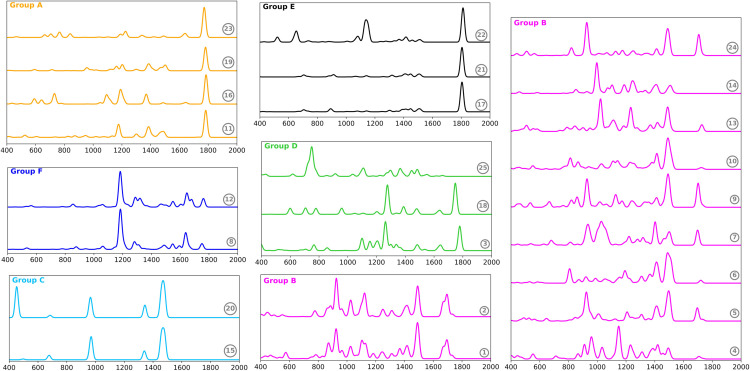
Classification of odorant compounds based on their infrared spectra. Each spectrum is labeled according to the compound numbers listed in Tables I and II. The spectra are color-coded by group to enhance visual distinction. Group A: 6-Methylhept-5-en-2-one, Benzoic acid, 3-Methyl-2-pentanone, Benzaldehyde. Group B: (E,E)-*α*-farnesene, (E)-*β*-ocimene, linalool, (-)-*β*-Caryophyllene, (-)-*α*-Copaene, Caryophylladienol II, *β*-Bisabolene, (E)-*α*-Bergamotene, (E)-Geranyl geraniol, Eucalyptol, d-Limonene. Group C: Dimethyl disulfide, Dimethyl trisulfide. Group D: methyl salicylate, acetophenone, indole. Group E: Octanal, Heptanal, Decanoic acid. Group F: (Z)-Methyl-p-methoxycinnamate, (E)-Methyl-p-methoxycinnamate.

Subsequently, the infrared spectra of odorant compounds were classified via the spectral clustering technique (Fig 2). Optimal results were attained with six clusters, as indicated by the Silhouette score. This metric was 0.48, indicating that the data are organized into distinctly separated groups. These groups are labeled with the letters A through F. The AMI score obtained was 0.44. This result suggests that odor categories are partially represented in the IR spectra, thereby supporting the notion that specific IR-active vibrational modes, often linked to functional groups, may play a role in odor discrimination [16,17]. Next, we describe the vibrational, structural, and olfactory characteristics of the compounds in each group.

Group A includes 6-methylhept-5-en-2-one, benzoic acid, 3-methyl-2-pentanone, and benzaldehyde. These compounds contain both carbonyl groups and C-C double bonds, and their odor is characterized by sweet and fruity notes (Table 1). The most prominent peak in their IR spectra corresponds to the carbonyl (C=O) stretching, which occurs below 1800 cm^−1^. Other observed bands are associated with the bending modes of CH, CH_2_, and CH_3_ groups. Structurally, compounds 23 and 16 are quite similar, as they are both aromatic carbonyl compounds, while compounds 11 and 19 are classified as aliphatic ketones.

Group B consists of terpenes with diverse aromas, including those of wood, flowers, fruits, and sweet notes [53,54]. These terpenes exhibit diverse geometries, with structures that can be acyclic, monocyclic, or bicyclic. The compounds of this group are predominantly found in the flowers of the Orchidaceae family. The infrared spectra present the following typical bands: out-of-plane bending =CH_2_ (900-950 cm^−1^), out-of-plane bending CH_2_ twisting and wagging (1000-1400 cm^−1^), in-plane bending CH_2_ scissoring (1450-1500 cm^−1^), and C=C stretching (1650-1700 cm^−1^). It is important to note that not all bands are present in every compound. Some compounds with similar odors share nearly the same IR spectrum within the group.

Group C consists of sulfur compounds, specifically dimethyl disulfide (DMDS) and dimethyl trisulfide (DMTS), both of which are known for their garlic odor. Their spectra display four distinct peaks: S–S stretching (452 cm^−1^), wagging of CH_3_ (963 cm^−1^), symmetric bending of CH_3_ (1344 cm^−1^), and asymmetric bending of CH_3_ (1470 cm^−1^). This particular set of peaks acts as a signature for these sulfur compounds, particularly at lower frequencies. Moreover, these compounds are consistently classified within the Apocynaceae family.

Group D comprises methyl salicylate, acetophenone, and indole. The first two compounds share significant vibrational, structural, and olfactory similarities, while indole can be categorized differently. Acetophenone and methyl salicylate are aromatic compounds that both contain a carbonyl group. They have a sweet scent, and their spectral analysis reveals two primary peaks related to carbonyl stretching (1750-1780 cm^−1^) and the typical stretching modes of aromatic ketones (C-C-C) and ethers (O-C-C). Conversely, indole displays a distinctive peak corresponding to out-of-plane C-H bending, which occurs around 750 cm^−1^. The remaining peaks, which have lower intensity, primarily correspond to C-C and C-N ring stretching. Additionally, indole has a notably unpleasant odor (decayed) and possesses a heterocyclic ring structure [41].

The compounds in Group E have a decayed odor and belong to the Apocynaceae family [55]. These include octanal, heptanal, and decanoic acid, all of which have a linear structure with the carbonyl group located at one end. Their IR spectra display a intense peak around 1800 cm^−1^, corresponding to the carbonyl group, while the other vibrational modes exhibit minimal intensity. Group F contains two isomers that emit a scent of anise (spices odor), which are odorants found in the flowers of the Orchidaceae family [56]. The vibrational mode with the highest intensity corresponds to the O–C–C asymmetrical stretch. Other peaks of lower intensity relate to C=C and C=O stretching (1600-1700 cm^−1^) and C-H bending (1200-1300 cm^−1^).

Overall, the classification of odorants based on their infrared spectra shows a consistent pattern related to their functional groups and odors. Compounds belonging to the same flower family tend to cluster together in the same group. Cis-trans isomers also form distinct groups. However, the most challenging group to classify was Group B, which includes compounds with differences in both aroma and IR spectra. The algorithm struggles to discriminate the subtle differences in their spectra. Nevertheless, there are similarities in their structure; all compounds are terpenes and share the isoprene unit.

### Raman spectra

We calculated the Raman spectra for the odorant compounds. Raman activity arises when a vibrational mode produces a change in the system’s polarizability, which is defined as the extent to which the electron cloud can be distorted by the electric field of the incident light [51]. First, we compare the theoretical and experimental spectra of three compounds: linalool, benzaldehyde, and (-)-*β*-caryophyllene (S2 Fig). Our calculations accurately match the profile of the experimental data. In particular, the double peaks corresponding to the two C=C stretching vibrations of caryophyllene and linalool are clearly observed in the theoretical spectrum. In addition, DFT calculations capture the relative intensity of the in-plane bending of CH_2_ in the structures, although they underestimate that associated with benzene ring bending. Thus, the theoretical spectra serve as a reliable reference for assessing the Raman activity of odorant compounds.

**Fig 3:**
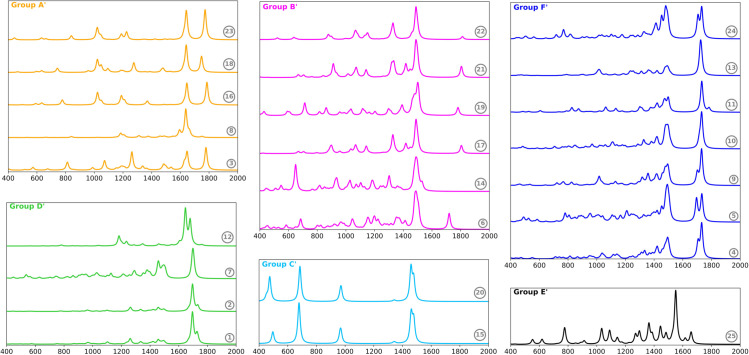
Classification of odorant compounds based on their Raman spectra. Each spectrum is labeled according to the compound numbers listed in Tables I and II. The spectra are color-coded by group to enhance visual distinction. Group A’: methyl salicylate, (Z)-Methyl-p-methoxycinnamate, Benzoic acid, Acetophenone, Benzaldehyde. Group B’: (-)-*α*-Copaene, Eucalyptol, Octanal, 3-Methyl-2-pentanone, Heptanal, Decanoic acid. Group C’: Dimethyl disulfide, Dimethyl trisulfide. Group D’: (E,E)-*α*-farnesene, (E)-*β*-ocimene, Caryophylladienol II, (E)-Methyl-p-methoxycinnamate. Group E’: Indole. Group F’: linalool, (-)-*β*-Caryophyllene, *β*-Bisabolene, (E)-*α*-Bergamotene, 6-Methylhept-5-en-2-one, (E)-Geranyl geraniol, d-Limonene.

Furthermore, we performed a classification of Raman spectra for the odorants via the spectral clustering method (Fig 3). For six clusters, we found a Silhouette coefficient of 0.51. This value is consistent with the qualitative similarity we observed in each group. The identified groups were labeled with A’ through F’. In addition, we obtained an AMI score of 0.33. This result indicates a weaker alignment between Raman-active vibrational features and odor categories, suggesting that vibrational modes based on polarizability capture less odor-relevant information than those active in IR within this dataset. In general, the clusters formed from the Raman spectra differ from those derived from the IR spectra. There is no group in the Raman clustering that is as large and diverse as group B in the IR, which could be considered a positive outcome. However, the methyl-p-methoxycinnamate isomers (compounds 8 and 12) are in a different group. This separation can be attributed to the polarizability of the stretching vibrational modes within the conjugated system.

Group A’ comprises benzenoid compounds that exhibit a sweet aroma. These odorants are found in both the Orchidaceae and Apocynaceae families (Fig 3). Their Raman spectra are characterized by a double band in the 1600 to 1800 cm^−1^ range, which corresponds to the C-C stretching of the conjugated system and the stretching of the carbonyl group. Peaks of lesser intensity include C-C ring bending, as well as in-plane C-H and O-H bending. In contrast, the compounds in group B’ show a greater diversity, including terpenes, aldehydes, aliphatic carboxylic acids, and a ketone. Octanal, heptanal, and decanoic acid possess a decayed aroma, while the other compounds can be categorized as wood and spices. Despite their structural and olfactory differences, the Raman spectra of these compounds show significant similarities. The most prominent peak corresponds to in-plane CH_2_ bending, while C=O and C=C stretching, along with other types of bending in the structure, display lower intensity.

The spectral clustering method identified the sulfur- and nitrogen-containing compounds in groups C’ and E’, respectively. These odorants are part of the Apocynaceae family. As previously mentioned, the indole has a fetid aroma, while the organosulfur compounds have a garlic scent. Raman spectra for Group C’ show four peaks, where DMDS and DMTS exhibit very similar spectra; however, DMTS has a higher intensity for the sulfur-sulfur (S-S) stretching. Group E’ consists exclusively of the indole, with the highest peak corresponding to C-C ring stretching at 1550 cm^−1^. Other representative bands are associated with ring-bending modes.

Groups D’ and F’ are predominantly found in the Orchidaceae family (Fig 3). The compounds in these two groups lack good odor discrimination. Each group exhibits a diverse range of aromas, including fruit, wood, spices, and flowers (Table 1). The Raman spectra of Group D’ show a band in the 1300-1500 cm^−1^ range, which has less intensity compared to Group F’. Group D’ mainly consists of terpenes, with the most prominent peak corresponding to the symmetric stretching of two C=C bonds, while a shoulder peak represents the C=C stretching of a single bond. In contrast, (E)-Methyl-p-methoxycinnamate is the compound that shows the least structural and Raman similarity. Group F’, on the other hand, presents some additional bands. The Raman spectra exhibit multiple active vibrations in the 1300-1500 cm^−1^ range, attributed to CH_2_ scissoring and CH_3_ symmetric bending.

Therefore, the classification based on Raman spectra reveals slight structural and odor similarities. Groups A’, C’, and E’ exhibited a strong correlation with the odor labels, while groups B’, D’, and F’ failed to discriminate the aroma effectively. This qualitative observation is consistent with the lower AMI score related to the clustering of IR spectra. Larger groups of compounds tend to mix molecules with varying odor descriptions. For instance, group B’, which contains six molecules, exhibits the most extensive diversity of odor descriptors. The most intense signal corresponds to in-plane CH_2_ bending, occurring around 1500 cm^−1^, which is not a distinctive vibrational mode. In contrast, some molecules, particularly those containing heteroatoms (such as sulfur and nitrogen) and extended conjugated systems like aromatic compounds, can be easily distinguished.

### Vibrational Density of States (VDOS)

**Fig 4:**
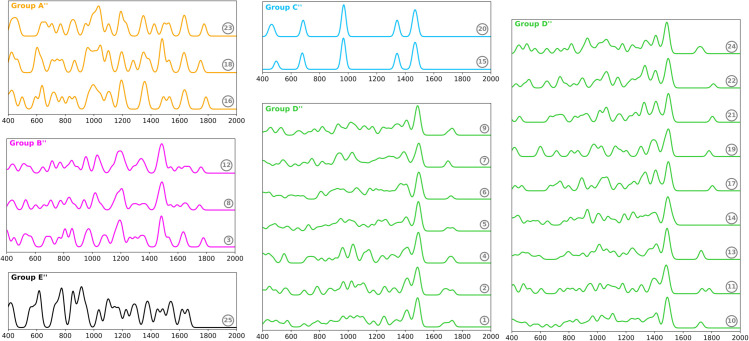
Classification of odorant compounds based on their Vibrational Density of States (VDOS). Each spectrum is labeled according to the compound numbers listed in Tables I and II. The spectra are color-coded by group to enhance visual distinction. Group A”: Benzoic acid, Acetophenone, Benzaldehyde. Group B”: methyl salicylate, (Z)-Methyl-p-methoxycinnamate, (E)-Methyl-p-methoxycinnamate. Group C”: Dimethyl disulfide, Dimethyl trisulfide. Group D”: (E,E)-*α*-farnesene, (E)-*β*-ocimene, linalool, (-)-*β*-Caryophyllene, (-)-*α*-Copaene, Caryophylladienol II, *β*-Bisabolene, (E)-*α*-Bergamotene, 6-Methylhept-5-en-2-one, (E)-Geranyl geraniol, Eucalyptol, Octanal, 3-Methyl-2-pentanone, Heptanal, Decanoic acid, d-Limonene. Group E”: Indole.

The vibrational density of states (VDOS) describes the distribution of vibrational modes per unit energy. This information is crucial for the analysis of the entire vibrational spectrum, as it can provide insight into the thermal and transport properties of a molecule. The Silhouette coefficient for the clustering analysis revealed that the optimal configuration consisted of five clusters (Fig 4). This metric was 0.57, indicating that the clustering is well-separated and compact in terms of geometric distance. The AMI value was equivalent to that obtained for Raman spectra (0.33), indicating a slight connection with the odor descriptors when considering all molecular vibrations.

Group D” includes sixteen compounds belonging to the Apocynaceae and Orchidaceae families, despite these molecules having different odors. This group exhibits peaks in the 1300-1600 cm^−1^ range, which are associated with the bending of CH, CH_2_, and CH_3_. At lower frequencies, bending modes of the carbon backbone appear similar. The only minor variation occurs in the 1600-1800 cm^−1^ region, which is associated with the stretching of C=C and C=O bonds. Consequently, the differences in VDOS are less pronounced compared to those observed in the Raman and IR spectra. However, the spectral profiles of Groups A”, B”, C”, and E” were distinctly different from each other. Groups A” and B” consist of aromatic carbonyl and aromatic ester compounds, respectively. The aroma of these molecules is sweet, resulting in analogous bands in their VDOS. Group C” identified the sulfur-containing compounds with similar peaks to those observed in the IR and Raman spectra. Finally, group E” comprises the indole, a bicyclic nitrogen heterocycle compound.

The VDOS clustering successfully categorizes certain compounds into four distinct groups based on their odor descriptors. However, the majority of molecules were grouped into a single cluster that contains a variety of odors. Consequently, while VDOS provides some valuable insights, it does not fully explain olfactory categorization. For example, the spectral clustering method fails to identify a band in the VDOS that distinguishes aliphatic aldehydes from terpenoids. Pandey et al. calculated the VDOS to classify the types of vibrational modes and rationalize the vibrational properties of 20 odorants. However, the pseudo-spectra between compounds with disparate odor characteristics exhibit numerous analogous bands, making this method less effective for differentiating between the odorants [20].

## Conclusion

In this work, we classified odorant compounds from two flower families using infrared spectra, Raman spectra, and vibrational density of states. Adjusted mutual information (AMI) scores revealed a significant association between vibrational spectra and odor descriptors, with infrared spectra providing the highest value. These results indicate that while the overall vibrational profile of each molecule does not align with perceptual odor, specific vibrational features are more closely related to the aroma [17]. Infrared spectroscopy, which emphasizes local characteristics such as functional group vibrations, provides more accurate odor classification compared to Raman spectroscopy and vibrational density of states.

Spectral clustering results can be interpreted by identifying specific and distinctive bands. In the IR spectra, terpenes present multiple peaks that correspond to the bending of CH_3_, CH_2_, and CH groups within their structures. In contrast, other compounds display considerable intensity for only a few bending modes. Another distinctive region is the C=C and C=O stretching, which is indicative of the functional groups. When an aromatic compound is present, in addition to the C=C stretching, a peak of similar intensity associated with out-of-plane C-H bending appears. In aliphatic chains, only the peak related to the carbonyl group is observed. In Raman spectra, higher intensity signals indicate symmetric vibrational modes, including ring bending, symmetric CH_3_ bending, and symmetric C=C stretches in conjugated systems. Finally, the analysis of the VDOS can be quite complex, as it encompasses all the vibrations of the system. Several peaks may be shared among molecules with different structures, functional groups, and odors. For instance, aliphatic aldehydes and terpenoids fall into the same group.

The clustering based on vibrational spectra groups together molecules whose molecular vibrations correspond to distinctive odors, such as garlic, decay, and sweetness. These odors are associated with compounds containing heteroatoms or conjugated systems. In addition, the highest AMI score for IR spectra clustering is consistent with previous studies that associate higher IR intensities with the inelastic electron tunneling mechanism [16,19]. This study directly contrasts IR and Raman spectroscopy (the latter being applied here for the first time in vibrational-odor research), thereby narrowing the physical phenomenology of odor and relating this characteristic more specifically to changes in dipole moment compared to changes in polarizability. Therefore, our findings suggest that vibrational spectra, particularly the infrared spectrum, can provide valuable insight for odor classification.

## Supporting information

S1 Fig Comparison between theoretical and experimental Infrared spectra of some odorant compounds. The theoretical spectra have been shifted by approximately 50 cm^−1^ towards lower frequencies.
(TIFF)

S2 Fig Comparison between theoretical and experimental Raman spectra of some odorant compounds. The theoretical spectra have been shifted by approximately 50 cm^−1^ towards lower frequencies.
(TIFF)

S3 Fig Molecular structures of odorant compounds identified in the flowers of the Apocynaceae and Orchidaceae families. Atom colors are coded as follows: carbon (gray), hydrogen (white), oxygen (red), nitrogen (blue), and sulfur (yellow). Each structure is labeled with the corresponding compound number as listed in Tables I and II.
(TIFF)

S4 Fig The similarity matrix for the infrared spectra of odorant compounds. (TIFF)

S5 Fig The similarity matrix for the Raman spectra of odorant compounds. (TIFF)

S6 Fig The similarity matrix for the Vibrational Density of States (VDOS) of odorant compounds. (TIFF)
