# Supplementary figures and images for: A comparative analysis of vibrational spectra for odorant classification
Source: PLoS One. 2026 Feb 26;21(2):e0342845. doi: 10.1371/journal.pone.0342845 (PMC12944807; doi:10.1371/journal.pone.0342845)

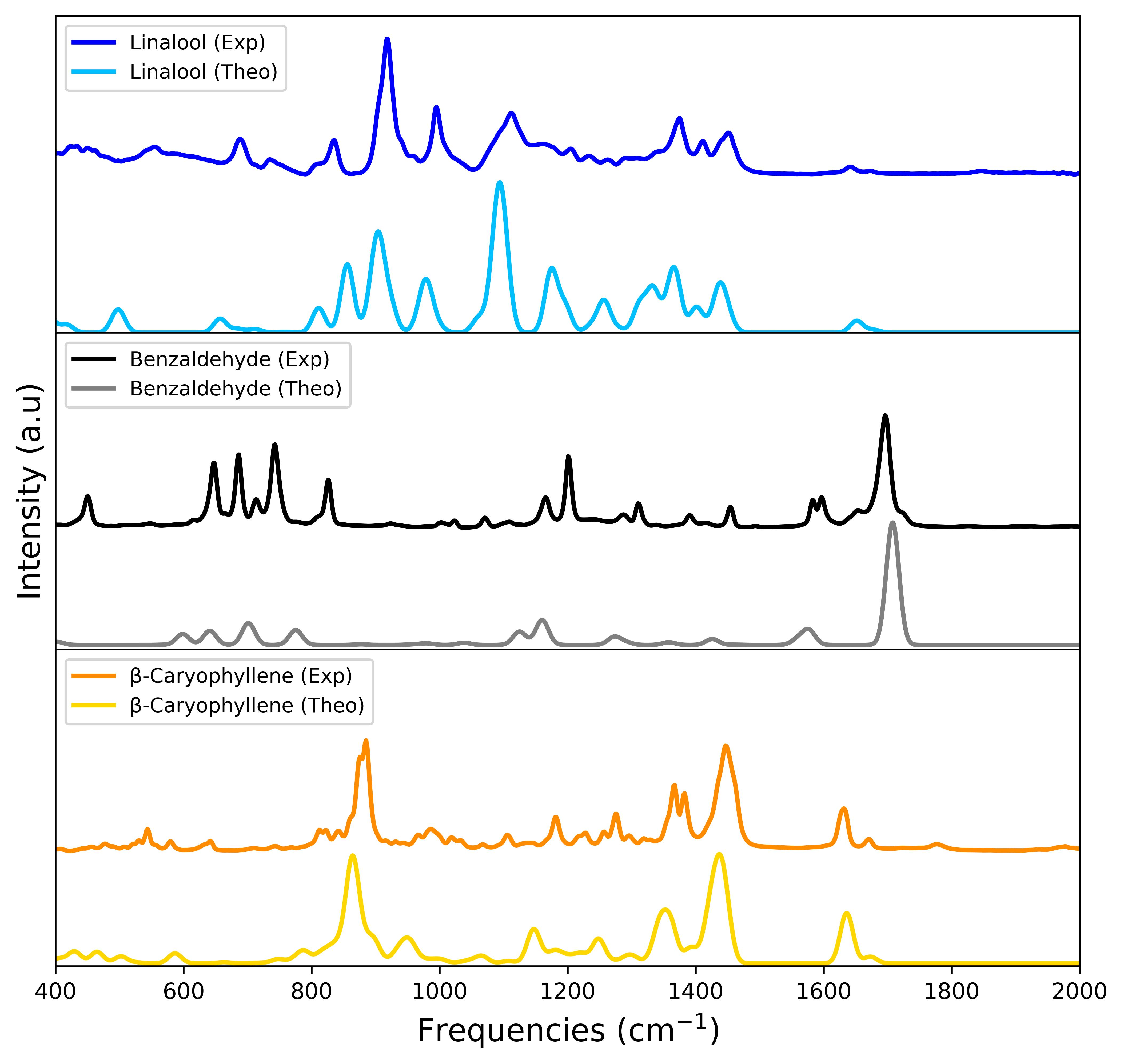

Supplement: S1 Fig — The theoretical spectra have been shifted by approximately 50 cm−1 towards lower frequencies. (TIFF) [file pone.0342845.s001.tiff]

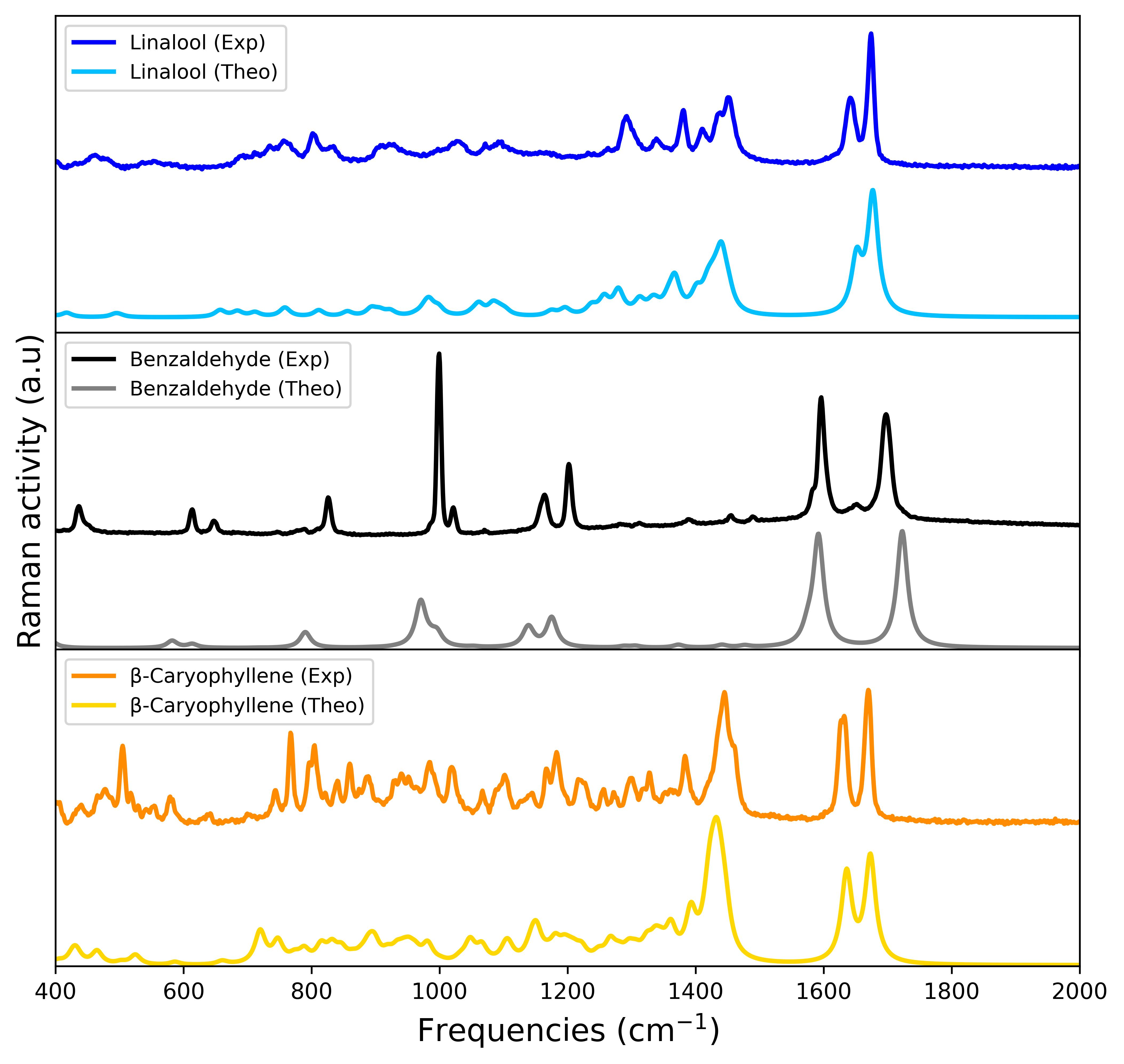

Supplement: S2 Fig — The theoretical spectra have been shifted by approximately 50 cm−1 towards lower frequencies. (TIFF) [file pone.0342845.s002.tiff]

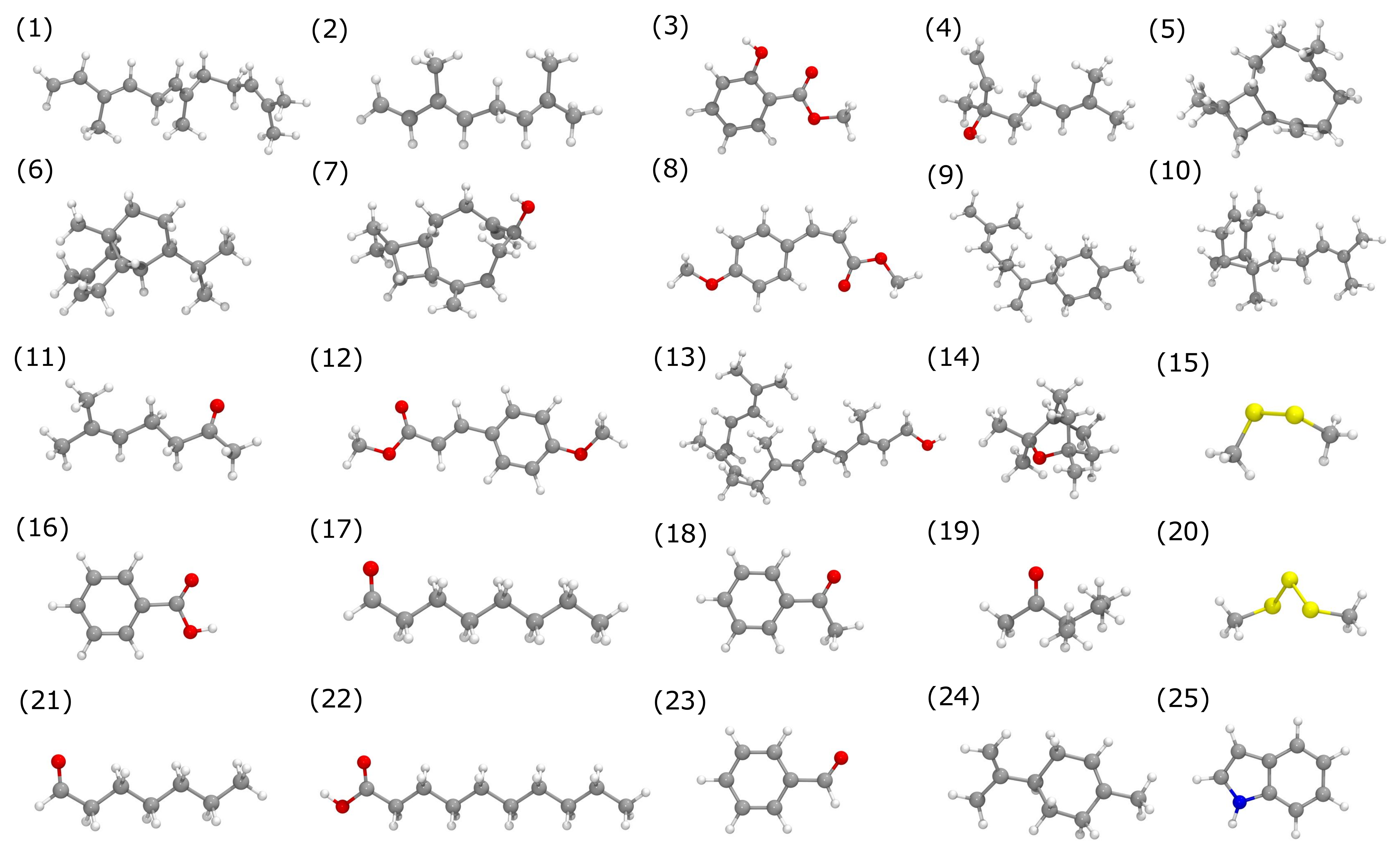

Supplement: S3 Fig — Atom colors are coded as follows: carbon (gray), hydrogen (white), oxygen (red), nitrogen (blue), and sulfur (yellow). Each structure is labeled with the corresponding compound number as listed in Tables I and II. (TIFF) [file pone.0342845.s003.tiff]

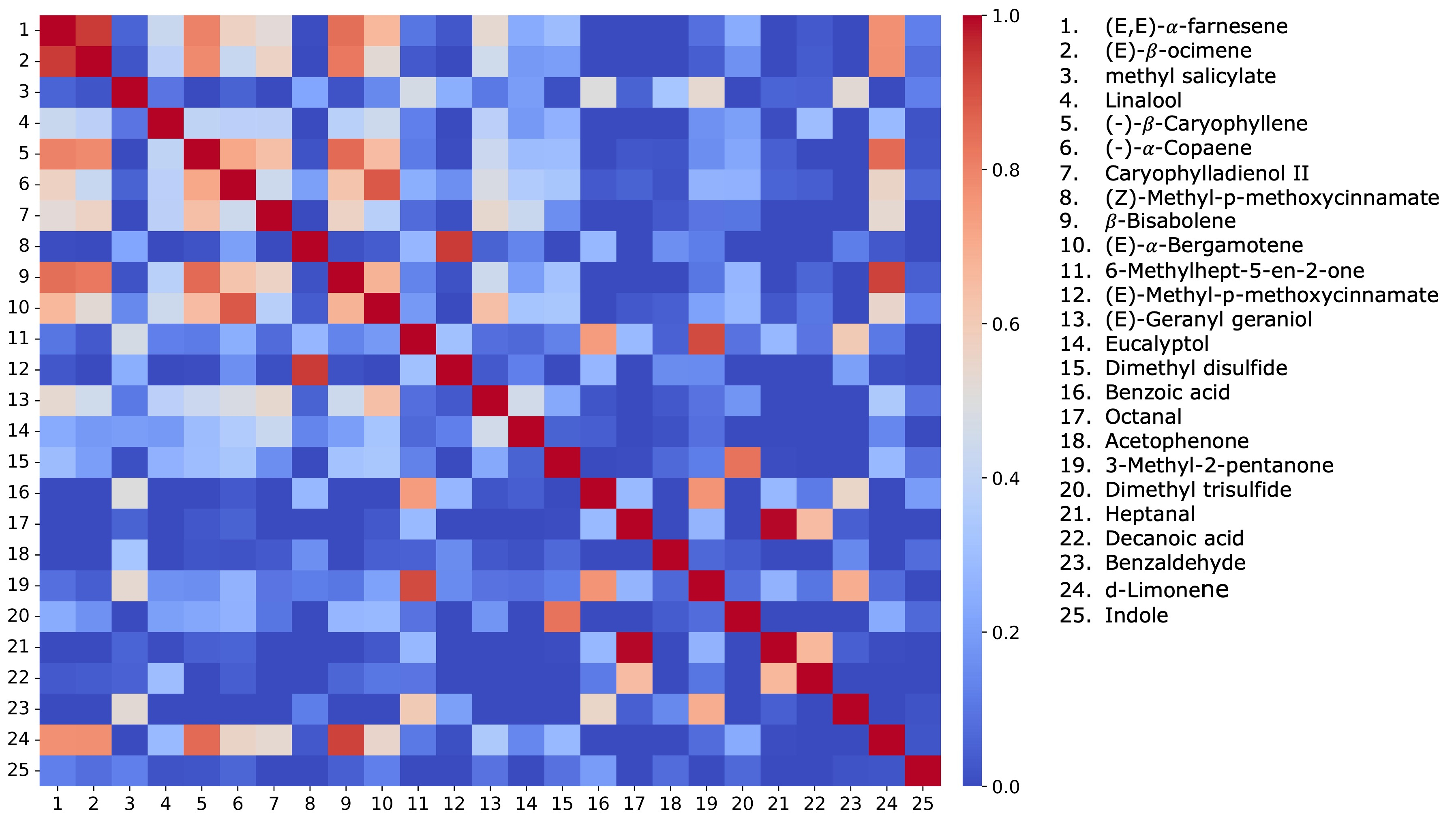

Supplement: S4 Fig — (TIFF) [file pone.0342845.s004.tiff]

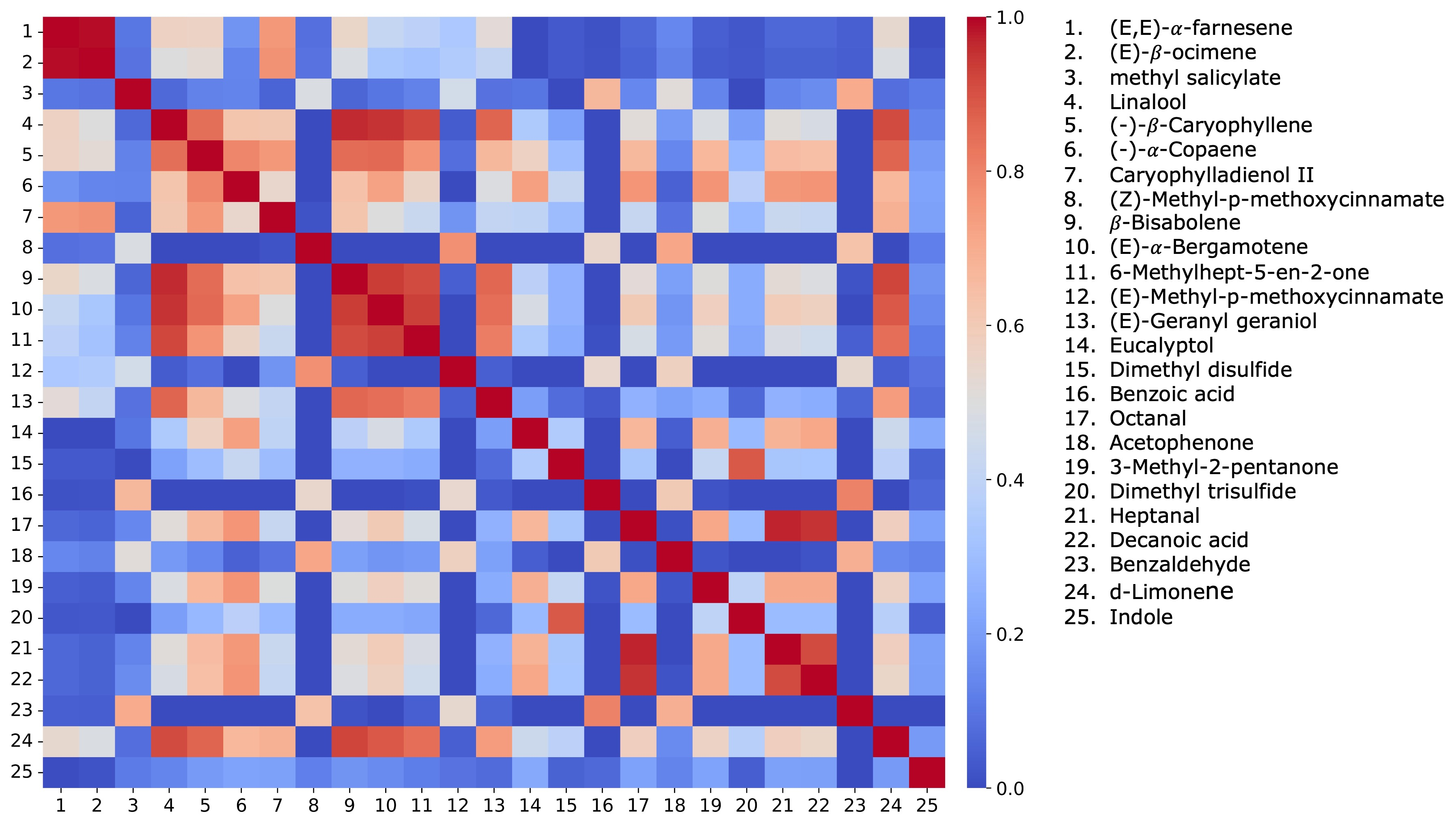

Supplement: S5 Fig — (TIFF) [file pone.0342845.s005.tiff]

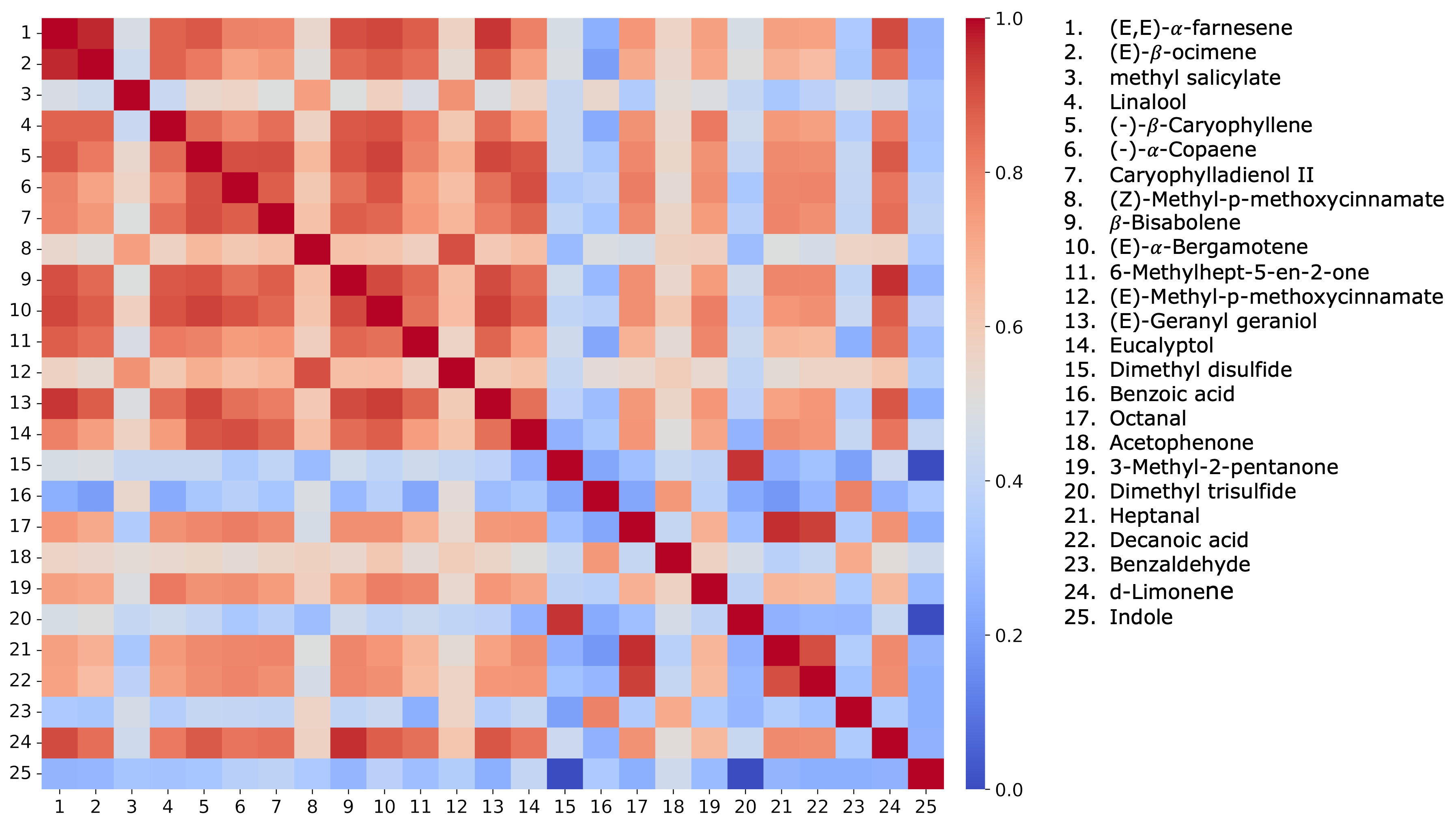

Supplement: S6 Fig — (TIFF) [file pone.0342845.s006.tiff]
